# Overexpression of Tyrosine hydroxylase and Dopa decarboxylase associated with pupal melanization in *Spodoptera exigua*

**DOI:** 10.1038/srep11273

**Published:** 2015-06-18

**Authors:** Sisi Liu, Mo Wang, Xianchun Li

**Affiliations:** 1 Department of Pesticide Science, College of Plant Science & Technology, Huazhong Agricultural University, Wuhan, 430070, China; 2 Department of Entomology and BIO5 Institute, University of Arizona, Tucson, AZ 85721, USA; 3 State Key Laboratory for Biology of Plant Diseases and Insect Pests, Institute of Plant Protection, Chinese Academy of Agricultural Sciences, Beijing, 100193, China; 4 Hubei Insect Resources Utilization and Sustainable Pest Management Key Laboratory, Institute of Insect Resources, Huazhong Agricultural University, Wuhan, 430070, China

## Abstract

Melanism has been found in a wide range of species, but the molecular mechanisms involved remain largely elusive. In this study, we studied the molecular mechanisms of the pupal melanism in *Spodoptera exigua*. The full length cDNA sequences of *tyrosine hydroxylase* (*TH*) and *dopa decarboxylase* (*DDC*), two key enzymes in the biosynthesis pathway of melanin, were cloned, and their temporal expression patterns in the integument were compared during the larval-pupal metamorphosis process of the *S. exigua* wild type (SEW) and melanic mutant (SEM) strains. No amino acid change in the protein sequence of TH and DDC was found between the two strains. Both *DDC* and *TH* were significantly over-expressed in the integument of the SEM strain at late-prepupa and 0 h pupa, respectively, compared with those of the SEW strain. Feeding 5^th^ instar larvae of SEM with diets incorporated with 1 mg/g of the DDC inhibitor L-α-Methyl-DOPA and 0.75 mg/g of the TH inhibitor 3-iodo-tyrosine (3-IT) resulted in 20% pupae with partially-rescued phenotype and 68.2% of pupae with partially- or fully-rescued phenotype, respectively. These results indicate that overexpressions of *TH* and *DDC* are involved in the pupal melanization of *S. exigua*.

Melanism, occurrence of dark (melanic) forms caused by over-deposition of the pigment melanin, is one of the most conspicuous phenotypic variations in external morphology. It has been documented in many different animal groups, including various insects^1^,^2^,^3^,^4^,^5^,^6^,^7^,^8^,^9^,^10^. In these documented insect species, the spatiotemporal expression profiles of melanization and the resultant advantages and fitness costs are usually different. For example, melanization is expressed locally in the two dipterans *Drosophila polymorpha*^4^ and *Drosophila immigrans*^6^, but globally in almost all the documented lepidopterans^2^,^3^,^9^,^10^,^11^,^12^,^13^,^14^,^15^,^16^,^17^,^18^. In terms of temporal expression pattern, melanic phenotype may express in larva^9^,^10^,^11^, pupa^14^, adult^2^,^3^,^16^,^19^, larva and adult (*Bombyx mori* mln mutant strain^20^), larva and pupa (*B. mori* sooty mutant strain^21^), or pupa and adult (*Helicoverpa armigera*^15^) stages. Further, melanism in these diverse taxa may confer one or more adaptive advantages^22^ that are associated with a fitness cost (e.g. in *Manduca sexta*^11^ and *H. armigera*^15^) or a fitness gain (e.g. in *Mythimna separate*^16^, *Spodoptera littoralis*^13^ and *D. immigrans*^6^) in life-history traits. Such variable expression profiles and adaptive impacts of melanization imply a great diversity of genetic/molecular mechanisms controlling melanism.

The genetic/molecular mechanisms underlying the great diversity of melanism in lepidopteran insects still remain largely a mystery. There are only three melanic mutant species (*B. mori*, *Papilio glaucus* and *M. sexta*) whose genetic/molecular bases of melanization have been elucidated by looking for alternations in the hormone cascades and/or the melanin and other pigment biosynthesis pathway genes. Among the two studied melanic mutants of *B. mori*, the *sooty* (*so*) mutant, which produces smoky larvae and black pupae, is caused by large deletions of the ORF (open reading frame) of the N-β-alanyltransferase (also known as N-β-alanyl dopamine synthase) gene *ebony*^21^, whereas the *melanism* (*mln*) mutant, in which melanization is expressed locally in larvae but globally in adults, is resulted from partial deletion of the 4^th^ exon of the *arylalkylamine-N- acetyltransferase* (*AANAT*) gene and its reduced expression^20^. Studies of the black larval mutant of *M. sexta* establish a causal link between JH (juvenile hormone) deficiency, higher DDC (dopa decarboxylase) expression, and the melanic phenotype^11^,^23^,^24^. However, such a link is not detected in the dark larval mutant of the butterfly, *Bicyclus anynana*^9^. In the melanic female adults of *P. glaucus*, local deposition of the melanin pigment on the wing is resulted from early suppression of N-β-alanyltransferase encoded by *ebony*, in concert with reduced DDC activity^25^,^26^,^27^. By contrast, the global industry melanization of the adults of the peppered moth (*Biston betularia*) is not linked to any of the sixteen melanin pathway genes identified so far^28^. As far as we know, there is no single case of pupal melanism, in which the underlying molecular mechanism has been elucidated.

To advance understanding of the molecular genetic bases of melanism, we choose to study the melanic mechanisms in a pupal melanic mutation strain (SEM) of the beet armyworm [*Spodoptera exigua* (Hübner)], which was established with 40 black pupae spontaneously occurred within a typical laboratory population (SEW, brown pupae) of *S. exigua*. The melanic phenotype is globally expressed only in the pupal stage. Relative to its parental wild-type strain SEW, the melanic SEM strain actually develops faster and has higher fecundity. The fact that melanin, rather than brown pigment as in the SEW strain, is synthesized/deposited in the newly-pupated SEM pupae (personal observation) suggest that alternations in one or more melanin- [(e.g. tyrosine hydroxylase (*TH* or *pale*) and DDC) or other pigments-promoting (e.g. *ebony* and *AANAT)* genes are probably responsible for the pupal melanic phenotype in the SEM strain. To test the melanin-promoting gene alternation hypothesis, here we compared the cDNA/protein sequences and temporal expression profiles of TH and DDC, two enzymes responsible for production of the melanin precursors, dopa and dopamine respectively, between the two strains. We also examined the *in vivo* effects of inhibiting TH and DDC on the melanic phenotype of the melanic SEM strain. We not only detected overexpression of *TH* and *DDC* in the integuments of SEM prepupae and puape, but also found that feeding larvae of the mutant SEM strain with inhibitors of TH and DDC led to conversion of the melanic pupae to wild type (brown) or intermediate (dark brown) color pupae. These results suggest that overexpression of the two melanin precursor-producing genes are responsible for the pupal melanic color morph in the SEM strain.

## Results

### TH cDNA in the wild type and melanic strains of *S. exigua*

**Figure 1:**
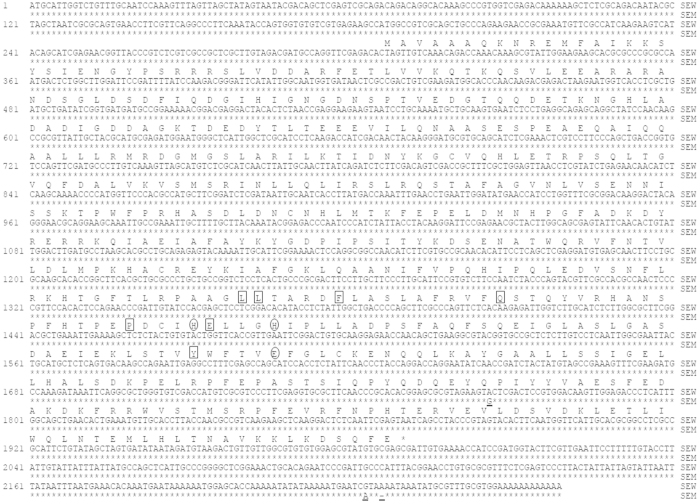
Alignment of TH cDNA sequences from the wild type (SEW) and melanic (SEM) strains of *S. exigua.* ○ = metal binding site of TH; □ = cofactor binding site of TH; — = SNP.

**Figure 2:**
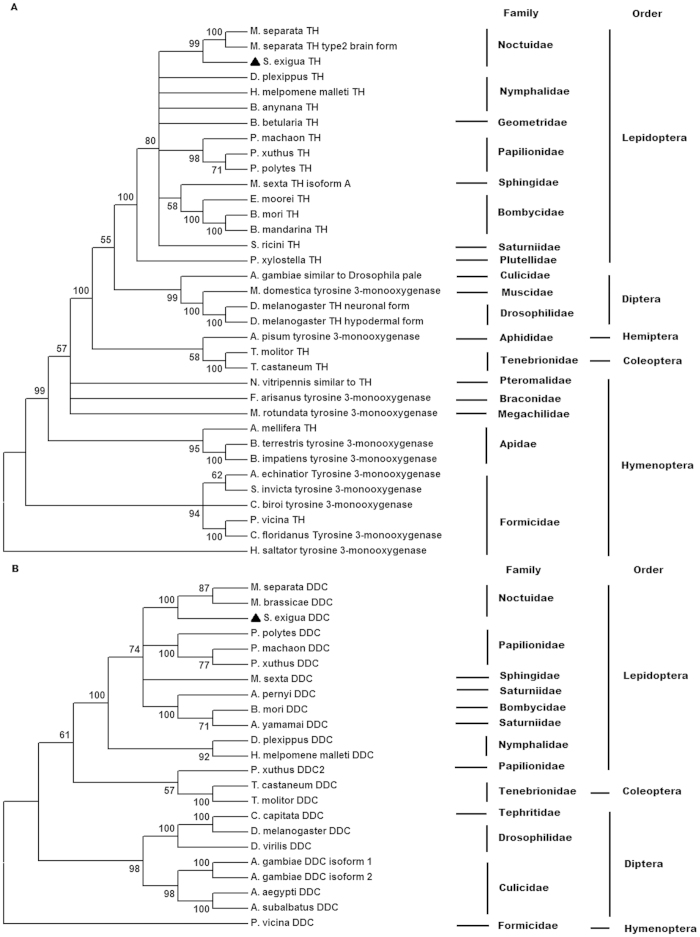
Phylogenetic trees of insect TH (A) and DDC (B) based on their amino acid sequences. Phylogenetic trees were constructed using the neighbor-joining method with 1000 bootstrap replicates. The numbers at each tree node are the bootstrap values.

The full-length cDNA sequences of *TH* and *DDC* from *S. exigua* were obtained by RT-PCR cloning of a partial fragment, followed by cloning of their 5’ and 3’ ends by 5’ and 3’ RACE (random amplification of cDNA ends). The NCBI accession numbers of the cDNA sequences of *TH* and *DDC* from SEW and SEM were JF795467, JF795468, JF795469 and JF795470, respectively. The *S. exigua TH* (*SeTH*) cDNA (2259 bp in total) contains a 185-bp 5’ UTR (untranslated region), an open reading frame (ORF) of 1686 bp encoding 561 amino acids, and a 386-bp 3’ UTR (Fig. 1). Sequence analysis shows that SeTH shares 97% amino acid identity with TH from *Mythimna separata*, 94% from *Papilio xuthus*, *M. sexta* and *B. mori*, 91% from *Heliconius melpomene malleti* and *Samia cynthia ricini*. Phylogenetic analysis also shows that SeTH is more closely related to TH from *M. separate,* which, like *S. exigua*, belongs to Noctuidae (Fig. 2A). Similar to the structure of other TH proteins, SeTH contains the conserved metal binding site for iron atom (the circled histidine 389, histidine 394 and glutamate 432 in Fig. 1) and the cofactor tetrahydrobiopterin (BH_4_) binding site (the boxed Leu251, leu252, phe257, gln267, pro385, glu390 and try429 in Fig. 1).

cDNA sequence alignment uncovers that the SeTH alleles from the wild type and melanic mutant strains only have 1 SNP (single nucleotide polymorphism) in the ORF, 1 SNP and 1 1-bp indel in the 3’ UTR (underlined in Fig. 1). The only SNP in the ORF does not cause amino acid change.

### DDC cDNA in the wild type and melanic strains of S. exigua

**Figure 3:**
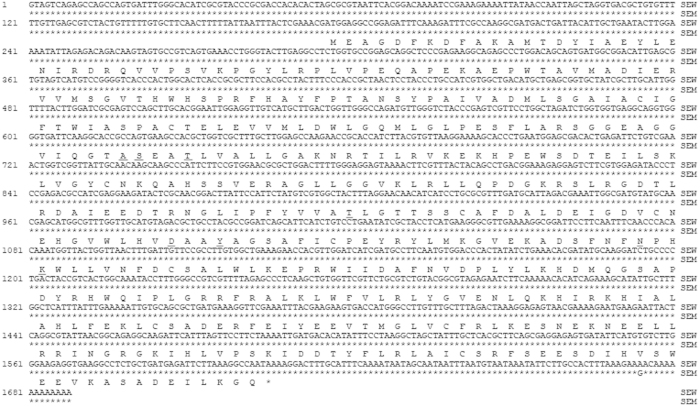
Alignment of DDC cDNA sequences from the wild type (SEW) and melanic (SEM) strains of *S. exigua*. — = cofactor binding site of DDC.

The *S. exigua DDC* (*SeDDC*) full-length cDNA is 1688 bp long, containing a 175-bp 5’UTR, an ORF of 1431 bp (from 176 to 1606) encoding 476 amino acids, and a 82-bp 3’UTR (Fig. 3). The predicted SeDDC proteins shows 95% amino acid sequence identity with *Mamestra brassicae* DDC, 94% with *M. separate* DDC, 91% with *Antheraea pernyi* and *Papilio machaon* DDC. SeDDC is grouped into the same lineage with *M. separata* and *M. brassicae* DDC, which share a common ancestor DDC with another lineage formed by the Papilionoidea DDC clades (Fig. 2B). Like other DDC, SeDDC has a cofactor binding site for pyridoxal 5’-phosphate (PLP). The PLP binding site is composed of ala148, ser149, thr152, thr242, asp271, try274, asn303 and lys306 (underlined in Fig. 3).

Only one nucleotide difference was found between the *DDC* cDNA sequences from SEW and SEM. This substitution occurs in the 3’UTR and thus causes no amino acid change between the two DDC alleles (Fig. 3).

### Overexpression of TH and DDC in the melanic SEM strain of *S. exigua*

**Figure 4:**
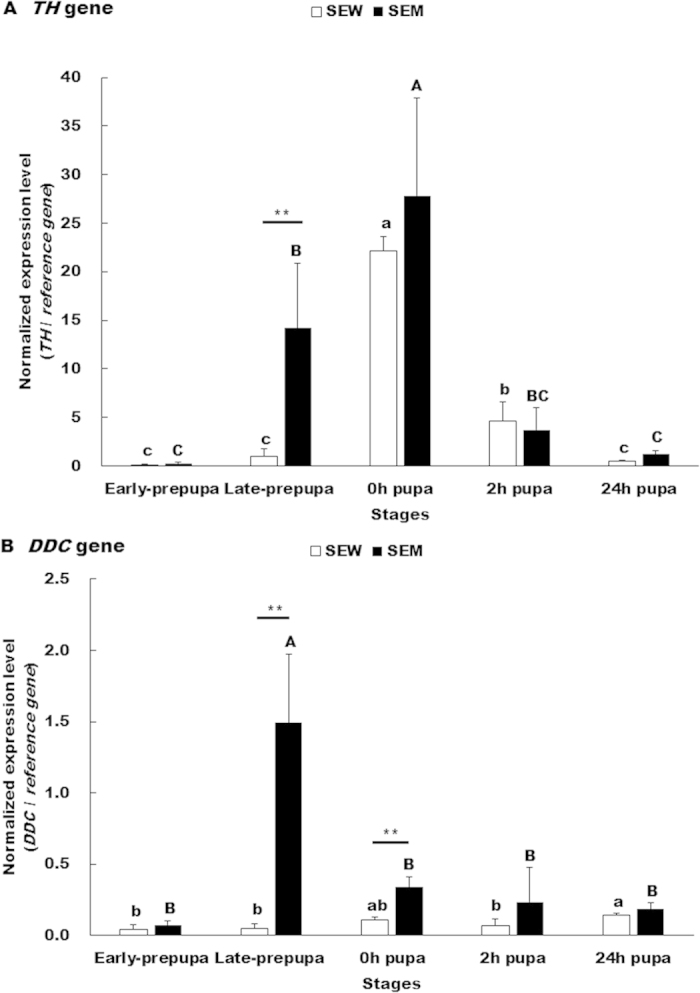
Transcriptional levels of *TH*(A) and *DDC* (B) in the wild type (SEW) and melanic (SEM) strains of *S. exigua*. The data and error bars represent the means and standard deviations of three (24 h pupae) or four (other 4 stages) biological replicates of three technical repeats each. Values sharing the same letter [small case letters for the wild type (SEW) strain, capital letters for the melanic (SEM) strain] are not significantly different at *P* < 0.05 (Tukey HSD tests). Significant differences at each of the five developmental stages between the two strains are indicated by one star (*, *P *< 0.05, paired *t*-test) and two stars (**, *P* < 0.01, paired *t*-test), respectively.

Two-way ANOVA revealed significant differences in *TH* expression between strains and among the five developmental stages (Table 1A). There were also significant strain × stage interactions in *TH* expression. The normalized expression of *TH* in the integument of the wild type SEW strain was in a descending order of 0-h pupae (22.134 folds) >2-h pupae (4.587 folds) >late prepupa (0.936 folds) >24-h pupae (0.517 folds) > early prepuape (0.136 folds) (Fig. 4A). The temporal expression file of *TH* slightly differed in the melanic mutant strain SEM, which had a descending order of 0-h pupae (27.820 folds) >late prepupa (14.219 folds) >2-h pupae (3.703 folds) >24-h pupae (1.190 folds) >early puape (0.272 folds). Relative to the wild type SEW strain, the expression of *TH* was significantly higher in the late prepupae (15.191 folds) of the SEM strain (Fig. 4A).

Two-way ANOVA also revealed significant differences in DDC expression between strains and among the five developmental stages as well as significant strain × stage interactions in DDC expression (Table 1B). Comparison of Fig. 4A,B revealed that *DDC* transcripts were much less abundant than *TH* transcripts in both the wild and mutant strains. The normalized expression of *DDC* in the integument of the wild type strain SEW was ranked as 24-h pupae (0.145 folds) >0-h pupae (0.110 folds) >2-h pupae (0.070 folds) >late prepupae (0.046 folds) >early prepupae (0.044 folds) (Fig. 4B). The temporal expression profile of *DDC* in the integument of the mutant SEM strain was ranked as late prepupae (1.492 folds) >0-h pupae (0.339 folds) >2-h pupae (0.235 folds) >24-h pupae (0.184 folds) >early prepupae (0.075 folds). However, *DDC* transcripts were significantly more abundant in SEM than in SEW in late prepupae (32.435 folds) and 0-h pupae (3.082 folds), respectively (Fig. 4B).

### Rescue of the mutant melanic phenotype by inhibiting TH and DDC

**Figure 5:**
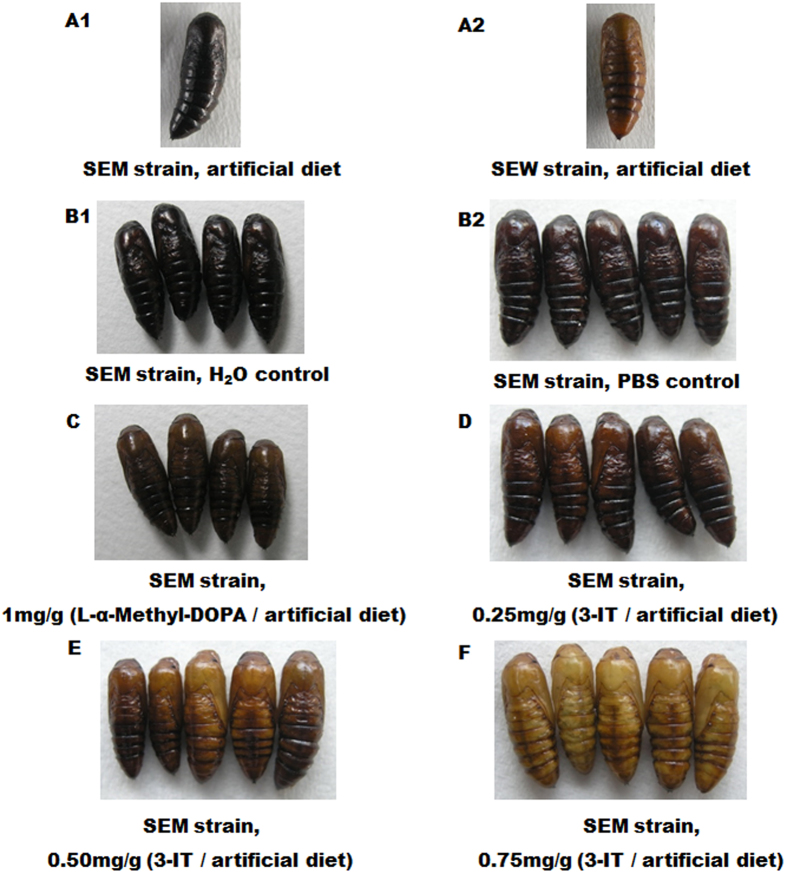
Phenotypes of the melanic SEM strain pupae pupated from larvae fed with diets containing the DDC inhibitor L-α-Methyl-DOPA or the TH inhibitor 3-IT.A1. Untreated SEM strain, 24 h old pupa; **A2**. Untreated SEW strain, 24 h old pupae; **B1**. SEM strain, H_2_O control, 5-day old pupae; **B2**. SEM strain, PBS control, 24 h old pupae; **C**. SEM strain, 1.0 mg L-α-Methyl-DOPA/g artificial diet, 5-day old pupae; **D**. SEM strain, 0.25 mg 3-IT/g artificial diet, 24 h old pupae; **E**. SEM strain, 0.50 mg 3-IT/g artificial diet, 24 h old pupae; **F**. SEM strain, 0.75 mg 3-IT/g artificial diet, 24 h old pupae.

To test if overexpression of TH and/or DDC is responsible for the mutant melanic phenotype in SEM, we fed 5^th^ instar larvae of SEM with control diets, diets incorporated with 0.25, 0.50, or 0.75 mg/g of 3-Iodo-L-tyrosine (3-IT, a TH inhibitor), or diets with 0.1, 0.5, and 1.0 mg/g of L-α-Methyl-DOPA (a DDC inhibitor). All the pupae obtained from larvae fed on control diets and diets with 0.1 or 0.5 mg/g of L-α-Methyl-DOPA were as black as the H_2_O control group (Fig. 5B1; Table 2), which were the same color as the SEM strain (Fig. 5A1), whereas 6 out of the 30 pupae pupated from larvae fed on diets with 1.0 mg/g of L-α-Methyl-DOPA were dark-brown (Fig. 5C; Table 2), i.e. partially rescued. The TH inhibitor 3-IT displayed even stronger rescue of the mutant phenotype. About half of the pupae from larvae fed on diet with 0.25 mg/g of 3-IT were partially recued and had a dark-brown color (Fig. 5D and Table 2), while all the pupae from the PBS control diets remained black (Fig. 5B2; Table 2). As the 3-IT dose increased, both the level of phenotype rescue (compare pupae in Fig. 5D-F) and the percentage of pupae with partially- (Fig. 5D,E) and fully-rescued (brown color pupae in Fig. 5F) simultaneously increased (Table 2), and the rescued phenotype is very close (Fig. 5E) and even lighter (Fig. 5F) than the wild-type morph (Fig. 5A2). The highest rescue rate was caused by 0.75 mg/g 3-IT (68.2%), which was not significantly higher than that elicited by 0.5 mg/g 3-IT (52.4%, *P *= 0.228) and 0.25 mg/g 3-IT (47.1%, *P *= 0.500). There was no significant difference between the rescue rates of 0.5 mg/g 3-IT and 0.25 mg/g 3-IT (*P *= 0.261).

## Discussion

The great diversity of insect melanism provides rich models for studying the molecular and developmental regulation of phenotypic variations. All the melanic mutant phenotypes, whether occurred locally or globally in space/time and associated with or without a fitness cost, are concentration of one or both of the two insect melanins—Dopa melanin and Dopamine melanin^20^,^28^,^29^—on a particular body surface at a particular developmental time. This can only happen if the precursors of one or both of the two melanins, Dopa and Dopamine respectively, are overproduced, or less of them are channeled to synthesize the yellow and/or colorless pigments in the melanic insect strains.

To test if one or both of the two melanin precursors are overproduced in the pupal melanic SEM strain of *S. exigua*, we contrasted the cDNA sequences and expression levels of TH and DDC, the two enzymes responsible for production of the Dopa and Dopamine precursors respectively, between the wild type SEW and melanic SEM strains. While we did not found any differences in the amino acid sequences of the two enzymes (Figs 1 and 3), we detected about 15-fold more *TH* transcripts in the early prepupae of SEM and more *DDC* transcripts in the late prepupae (32.435 folds) and 0-h pupae (3.082 folds) of SEM (Fig. 4). The fact that inhibiting the overproduced TH and DDC in SEM resulted in fully- and partially-rescued pupae respectively (Table 2 and Fig. 5) demonstrates that overproduction of TH and DDC is responsible for the pupal melanism in the SEM strain of *S. exigua.* Whether less consumption of the Dopa and Dopamine precursors for synthesis of the yellow and colorless pigments also contributes to SEM’s melanic phenotype has yet to be determined.

As far as we know, our study is the first report of molecular mechanisms underlying melanism occurred only in the pupal stage of lepidoperan insects. Among the limited cases studying the mechanisms of lepidoperan melanism, 2 involved melanization in the larval stage^9^,^24^, 2 in the adult stage^26^,^27^,^28^, 1 in the larval and adult stage^20^, and 1 in the larval and pupal stage^21^. Overproduction of the Dopamine precursor due to enhanced *DDC* expression is linked to the larval melanism of *M. sexta*^24^. By contrast, less consumption of the Dopa precursor for synthesis of the yellow pigment by simultaneously-reduced expression of *ebony* and *DDC* causes partial melanization on the female adult wing of *P. glaucus*^25^,^26^,^27^. Similarly, less consumption of the Dopamine precursor for synthesis of the yellow or colorless pigment by deletion of the ebony ORF or the 4^th^ exon of *AANAT* is linked to the *so* (larval and pupal melanization^21^) and *mln* (larval and adult melanization^20^) mutants of *B. mori*, respectively. Thus, our finding of TH overexpression as the major contributor to the pupal melanism of *S. exigua* also represents the first report of the involvement of TH in insect melanism mutation. This finding is consistent with an earlier report that reduced expression of TH is linked to the light neonate color in the sex-linked *chocolate* (*sch*) mutant of *B. mori*^30^.

While we have discovered that overexpression of TH and DDC is responsible for the pupal melanization in the SEM strain of *S. exigua*, we have not identified the actual alterations that lead to increased expression of the two genes. One possibility would be an alternation in a transcription factor or small RNA that regulates expression of both *TH* and *DDC*. Another possibility would be some indel or SNP mutations in the 5’ promoter regions of both genes that separately enhance expression of the two genes. The third possibility would be an alternation in the promoter region of TH or its transcription factor/small RNA that only increases TH expression, but the resultant extra Dopa precursor induces the expression of the downstream DDC gene. Clearly, molecular cloning of the promoter regions of the two genes is the first step needed to resolve these speculations.

## Methods

### Experimental animals

The wild-type strain (SEW, brown color pupae) of *S. exigua* was established with a field collection from Jingzhou, Hubei, China in 2003 and has been maintained on artificial diet (a solid mixture of soybean flour, barley flour, yeast powder, Vitamin C, benzoic acid, sodium benzoate, acetic acid and agar) at 27 ± 1 °C, 65% relative humility and a photoperiod of 14:10 (L: D). At the 6^th^ generation, forty black pupae spontaneously occurred within the SEW strain. The adults emerged from the 40 black pupae were crossed to establish the pupal melanic strain (named SEM). The SEM strain has then been maintained on the same artificial diet at the same conditions with the SEW strain since then.

### TH and DDC cDNA Cloning

For each strain, three prepupa integuments were dissected out on a filter paper placed on ice, washed with cold 0.1% DEPC-treated water, briefly dried on a clean filter paper, transferred to a ceramic mortar with liquid nitrogen, and ground to powder in liquid nitrogen. The resultant prepupa intergument power of each strain was immediately transferred into a 1.5-mL centrifuge tube with about 500 μL of Trizol^TM^ reagent (Invitrogen, USA). Total RNA was isolated from the power according to the manufacturer’s instructions. RNA purity was evaluated by 260/280 and 260/230 ratios measured using NanoDrop 1000 (NanoDrop, USA). DNase I (Fermentas, EU) treated RNA (1 μg) was reverse transcribed into cDNA in a 20 μL reaction with random primer (N9) and Oligo dT(18) primer using First-Strand cDNA Synthesis Kit (Fermentas, EU).

The primers for cloning of *TH* partial cDNA (Table S1) were designed based on the conserved regions of tyrosine hydroxylases of insect. The PCR conditions were 3 min denaturation at 95 °C, followed by 40 cycles of 30 sec denaturation at 94 °C, 30 sec annealing at 50 °C and 1 min extension at 72 °C, and a final 9 min extension at 72 °C. The primers for cloning of *DDC* partial cDNA (Table S1) were designed on the basis of *S. exigua* dopa decarboxylase (*DDC*) gene (NCBI accession no.: SEU71404). The PCR conditions were 3 min denaturation at 95 °C, followed by 40 cycles of 30 sec denaturation at 94 °C, 30 sec annealing at 56 °C and 30 sec extension at 72 °C, and a final 9 min extension at 72 °C. The full-length cDNAs of the two genes were obtained by rapid amplification of cDNA ends (RACE) technique using the 3’-Full RACE Core Set Ver. 2.0 (TaKaRa, Japan) and 5’-Full RACE Kit (TaKaRa, Japan) with the corresponding specific primers (Table S1) designed based on their partial cDNA sequences. The open reading frame (ORF) of *TH* and *DDC* were amplified from SEW and SEM strains with the corresponding flanking PCR primers (Table S1), respectively. The flanking PCR conditions for *TH* gene was 3 min denaturation at 95 °C, followed by 40 cycles of 30 sec denaturation at 94 °C, 30 sec annealing at 54 °C and 2 min extension at 72 °C, and a final 9 min extension at 72 °C. The flanking PCR conditions for *DDC* gene was 3 min denaturation at 95 °C, followed by 40 cycles of 30 sec denaturation at 94 °C, 30 sec annealing at 50 °C and 2 min extension at 72 °C, and a final 9 min extension at 72 °C. All the fragments and full ORF were cloned into pMD18-T Vector (TaKaRa, Japan) and at least three clones were sequenced for each fragment or ORF from the two strains.

### Sequence alignment and phylogenetic analysis

The nucleotide and deduced amino acid sequences of TH and DDC were aligned to identify potential changes between the wild type (SEW) and mutant (SEM) strains. The conserved domains of the deduced protein sequences were localized by blast against the conversed domain database (CDD) ( http://blast.ncbi.nlm.nih.gov/). The protein sequences retrieved from NCBI database and used for phylogenetic analysis were TH from the two strains of *S. exigua* (NCBI accession no.: AFG25778.1, AFG25779.1), *Drosophila melanogaster* (AAA62877 and AAA62876), *Musca domestica* (XP_005180802.1), *M. sexta* (ABQ95973.1), *Tribolium castaneum* (ABQ95974.1), *Tenebrio molitor* (ACU77882.2), *H. melpomene malleti* (ADU32895.1), *Bombyx mandarina* (ADV56709.1), *M. separata* (BAF32573.1 and BAF32574.1), *S. ricini* (BAF64534.1), *B. mori* (BAH11148.1), *Apis mellifera* (NP_001011633.1), *Ernolatia moorei* (ADV32917.1), *Harpegnathos saltator* (XP_011137576.1), *Fopius arisanus* (XP_011299399.1), *Cerapachys biroi* (XP_011336847.1|), *Solenopsis invicta* (XP_011157658.1), *Megachile rotundata* (XP_003704650.1), *Bombus terrestris* (XP_003396906.1), *Bombus impatiens* (XP_003486644.1), *Bicyclus anynana* (AGT62463.1), *Anopheles gambiae* (EAA44132), *Acromyrmex echinatior* (EGI66597.1), *Nasonia vitripennis* (XP_008203627.1), *Acyrthosiphon pisum* (XP_008182999.1), *Camponotus floridanus* (EFN71001.1), *Polyrhachis vicina* (AEC14314.1), *Danaus plexippus* (EHJ75630.1), *Plutella xylostella* (ADK12633.1), *Biston betularia* (ADF43213.1), *Papilio polytes* (BAJ07593.1), *P. xuthus* (BAE43824.1) and *P. machaon* (BAJ07587.1) as well as DDC from the two strains of *S. exigua* (AFG25780.1, AFG25781.1), *M. brassicae* (BAB68545.1), *M. separata* (BAB68549.1), *A. pernyi* (AAR23825.1), *Antheraea yamamai* (AAR23824.1), *P. machaon* (BAJ07588.1), *Danaus plexippus* (EHJ63554.1), *M. sexta* (AAC46604.1), *P. xuthus* (BAE43825.2, BAM18274.1), *Papilio polytes* (BAJ07594.1), *B. mori* (NP_001037174.1), *D. melanogaster* (P05031.4), *D. virilis* (EDW57710.1), *Armigeres subalbatus* (AAT75222.1), *A. gambiae* (AAC16249.1 and AAC16247.1), *Aedes aegypti* (AAC31639.1), *Armigeres subalbatus* (AAT75222), *Ceratitis capitata* (CAA69668.1), *Polyrhachis vicina* (AFI80897.1), *T. castaneum* (ABU25222.1), *T. molitor* (BAA95568.1) and *H. melpomene malleti* (ADU32894.1). Phylogenetic trees were constructed with the MEGA 4 program, using the neighbor-joining method. The confidence of the various phylogenetic lineages was assessed by the bootstrap analysis (bootstrap = 1000). The phylogenetic tree of TH was rooted by using the sequence of *Harpegnathos saltator* from Hymenoptera, and the phylogenetic tree of DDC was rooted by using the sequence of *Polyrhachis vicina* from Hymenoptera^31^.

### Real-time RT-PCR analysis of *TH* and *DDC* expression

Total RNA was isolated from the dissected integuments from both strains at five time points (early prepupa, late prepupa, 0 h pupa, 2 h pupa, 24 h pupa) and reverse transcribed into cDNA as described above. The cDNAs were used as the templates for quantitative RT-PCR (qRTPCR) analysis of *TH* and *DDC* expression using SYBR^®^ Premix Ex Taq^TM^ II kit (TaKaRa, Japan). Real-time PCR of *TH*, *DDC*, *β-actin* and *GAPDH* (*β-actin* and *GAPDH* were internal reference genes) were performed individually in a 20 μL reaction containing 2×SYBR® Premix Ex Taq^TM^ II 10 μL, 10 *μ*M forward primer and reverse primer (0.8 μL each), 1 μL template cDNA, and 7.4 μL nuclease-free water. Specific primers (Table S1) were designed for real-time PCR of *TH*, *DDC, β-actin* and *GAPDH* to generate the corresponding amplicons of 142 bp, 151 bp, 107 bp and 174 bp, respectively. All real-time PCR reactions were performed in iQ™ 96-well PCR plates (Bio-Rad, USA). A CFX96 real-time PCR detection system (Bio-Rad, USA) was used as the fluorescence detector with the following common PCR conditions for the three genes: an initial denaturing cycle of 95 °C for 30 s, followed by 40 cycles of denaturation at 95 °C for 5 s, annealing at 60 °C for 10 s and extension at 72 °C for 30 s, and data collection and real-time analysis enabled at 72 °C. Melting curve analysis from 65 °C to 95 °C was run for each target and reference gene to ensure free of junk products. For the time points of early prepupae, late prepupae, 0 h pupa and 2 h pupa, there were 4 biological replicates of 3 integuments each for each strain for each gene; for the time point of 24 h pupa, 3 biological replicates of 3 integuments each for each strain for each gene were tested; each biological replicate was qRT-PCR-analyzed 3 times. The expression level of all the genes were calculated with their mean Ct and amplification efficiency (*E*) (Equation 1), and the expression level of the two target genes (*TH* and *DDC*) at each time point of each strain were normalized with the geometric mean of the expression of the two reference genes (*β-actin* and *GAPDH*) (Equation 2)^32^,^33^.


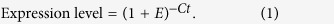


Normalized expression level of target gene


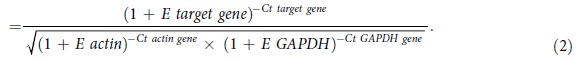


### Enzyme inhibitor treatment

The TH inhibitor 3-iodo-tyrosine (3-IT) (Sigma) PBS solution was incorporated into artificial diets at the final concentrations of 0.75, 0.50 and 0.25 mg/g diets according to previous studies^30^,^34^. The DDC inhibitor L-α-Methyl-DOPA (Sigma) water solution was mixed with artificial diets at 1.0, 0.5, and 0.1 mg/g diets according to a previous study^35^. The control diets for 3-IT and L-α-Methyl-DOPA were diets containing equal volume of PBS solution and water, respectively. For each inhibitor or control diets, 40 newly molted 5^th^ instar larvae of the melanic SEM strain were fed with the corresponding diets till pupation. The body colors of the SEM pupae were observed and photos were taken with a digital camera (OLYMPUS C5060) under white light. The numbers of pupae and adults obtained were recorded at the end of this experiment. The black pupae resulted from this inhibitor feeding experiment were considered as mutant melanic pupae, whereas the dark-brown and brown pupae obtained were considered as partially- and fully-rescued wild type pupae, respectively. For each concentration of inhibitor, the numbers of melanic, partially-rescued, and fully-rescued pupae were recorded and the rescue rate was calculated using the formula of the number of partially- and fully-rescued pupae/the total number of pupae ×100%.

### Statistical analysis

Two-way ANOVA was performed to examine the impacts of strain, stage and strain × stage interaction on the expressions of *TH* and *DDC*. Paired *t*-tests were conducted to test the significance of differences in the expression levels of *TH* and *DDC* at each of the five developmental stages between the two strains. Tukey HSD tests were performed to test the significance of differences in the expression levels of *TH* and *DDC* among the five developmental stages of each strain. The differences in the rescue rate among different concentrations of 3-IT were analyzed with one-tailed Fisher’s exact test. All of the statistical tests were performed by SPSS version 16.0 software for Windows.

## Additional Information

**How to cite this article**: Liu, S. *et al.* Overexpression of Tyrosine hydroxylase and Dopa decarboxylase associated with pupal melanization in *Spodoptera exigua*. *Sci. Rep.* **5**, 11273; doi: 10.1038/srep11273 (2015).

## Supplementary Material

Supplementary Information

## Figures and Tables

**Table 1:** Two-way ANOVA on the expressions of *TH* and *DDC* at different development stages between SEW and SEM strains of *S. exigua*

A. Two-way ANOVA analysis results of *TH* gene					
Source of variation	df	SS	MS	F	P
Strain	1	133.883	133.883	7.857	0.009
Stage	4	3218.326	804.582	47.219	<0.0001
Strain × Stage Interaction	4	272.158	68.039	3.993	0.011
Error	28	477.097	17.039		
Total	38	6489.238			
B. Two-way ANOVA analysis results of *DDC* gene					
Source of variation	df	SS	MS	F	P
Strain	1	1.367	1.367	42.684	<0.0001
Stage	4	2.537	0.634	19.799	<0.0001
Strain × Stage Interaction	4	2.828	0.707	22.070	<0.0001
Error	28	0.897	0.032		
Total	38	10.755			

**Table 2:** Statistics of pupae color changes resulted from feeding larvae of the melanic SEM strain of *S. exigua* with TH and DDC inhibitors.

Inhibitor	Concentration (mg/g)	Total number of pupae	Number of pupae with the following color			Rescue rate^1^ (%)
			Black	dark-brown	brown	
L-α-Methyl-DOPA	1	30	24	6	0	20.0
	0.5	25	25	0	0	0.0
	0.1	29	29	0	0	0.0
	CK (H_2_O)	28	28	0	0	0.0
3-IT	0.75	22	7	6	9	68.2
	0.50	21	10	6	5	52.4
	0.25	17	9	8	0	47.1
	CK (PBS)	16	16	0	0	0.0

^1^Rescue rate =(the number of dark-brown and brown pupae/the total number of pupae) × 100%.
