# Supplementary material for: Overexpression of Tyrosine hydroxylase and Dopa decarboxylase associated with pupal melanization in Spodoptera exigua
Source: Sci Rep. 2015 Jun 18;5:11273. doi: 10.1038/srep11273 (PMC4471665; doi:10.1038/srep11273)
Supplement: Supplementary Information [file srep11273-s1.doc]

**Overexpression of Tyrosine hydroxylase and Dopa decarboxylase are associated with pupal melanic mutation in *Spodoptera exigua***

Sisi Liu^1,2,4^, Mo Wang^1,4^*, Xianchun Li^2,3^*

^1^Department of Pesticide Science, College of Plant Sciences & Technology, Huazhong Agricultural University, Wuhan, 430070, China.

^2^Department of Entomology and BIO5 Institute, University of Arizona, Tucson, AZ 85721, USA.

^3^State Key Laboratory for Biology of Plant Diseases and Insect Pests, Institute of Plant Protection, Chinese Academy of Agricultural Sciences, Beijing, 100193, China.

^4^Hubei Insect Resources Utilization and Sustainable Pest Management Key Laboratory, Institute of Insect Resources, Huazhong Agricultural University, Wuhan, 430070, China.

Correspondence: Xianchun Li, 1140 E. South Campus Dr., Department of Entomology, University of Arizona, Tucson, AZ 85721, U.S.A. E-mail: lxc@email.arizona.edu; Mo Wang, 1 Shizishan St., Department of Pesticide Science, College of Plant Science & Technology, Huazhong Agricultural University, Wuhan, Hubei 430070, China. E-mail: wangmo@mail.hzau.edu.cn

**Supplementary table 1. Primers used for cloning and expression analysis of *S. exigua* *TH* and *DDC***

| Primer | Sequence (5’-3’) | Efficiency |
| --- | --- | --- |
| cDNA Cloning  (RT-PCR) |  |  |
| *TH* partial cDNA |  |  |
| Sense | GCGAAATGTTCGCCATCA | N.A. |
| Antisense | CARCRGGTTCDGGDGTRTGGA |  |
| *DDC* partial cDNA |  |  |
| Sense | TGAATGGAGCGACACTGAG | N.A. |
| Antisense | TTTCAGCCACAAGGCGGAACAAT |  |
| 5’ and 3’ RACE |  |  |
| *TH* |  |  |
| 5’GSP1 | CGGTTAGAGTGTAGTCCTCGTCC | N.A. |
| 5’GSP2 | TATCAGCATCAGCGAGGTGACCATTCTTAG |  |
| 3’GSP1 | AACACTGTATTGGACTTGATGCCT | N.A. |
| 3’GSP2 | TTCCGTGTCTTCCAATCTACCC |  |
| *DDC* |  |  |
| 5’GSP1 | TGTTCCCAACGTAGCCACGAC | N.A. |
| 5’GSP2 | AATCTCAGTGTCGCTCCATTCAGGGTGC |  |
| 3’GSP1 | GGTGGTGAGGCAGGTGGGGTGATTC | N.A. |
| 3’GSP2 | ATTCTATGTCGT GGCTACTTTGG |  |
| ORF flanking PCR |  |  |
| TH |  |  |
| Sense | ATGCATTGGTCTGTTTGCAATCCAAAGTTT | N.A. |
| Antisense | CCACACACGCCAACAACAGT |  |
| DDC |  |  |
| Sense | TTATAACCAATTAGCTAGGTGACGC | N.A. |
| Antisense | GCTATTATTTTGAAATGCAAAGTCC |  |
| Expression analysis (real-time PCR) |  |  |
| *TH* |  |  |
| Forward | ACGCCGTCAAGAAGCTCAAG | 91.4% |
| Reverse | CCACACACGCCAACAACAGT |  |
| *DDC* |  |  |
| Forward | GAAAGAACCACGTTGGATCATCG | 98.6% |
| Reverse | CAGACGCAGAACGAACCACAG |  |
| *β-actin* ^36^ |  |  |
| Forward | ATCCTCCGTCTGGACTTGG | 93.4% |
| Reverse  GAPDH ^37^  Forward  Reverse | CGCACGATTTCCCTCTCA  GACAACCACTCATCTATCTTCG  AACATTTATCTCTACAACGCAATC | 90.0% |

N.A.: Not applicable
